# Association between Triplex-Forming Sites of Cardiac Long Noncoding RNA *GATA6-AS1* and Chromatin Organization

**DOI:** 10.3390/ncrna8030041

**Published:** 2022-06-01

**Authors:** Benjamin Soibam

**Affiliations:** Computer Science and Engineering Technology, University of Houston-Downtown, Houston, TX 77002, USA; soibamb@uhd.edu; Tel.: +1-713-226-5216

**Keywords:** *GATA6-AS1*, CTCF, topologically associated domains, RNA–DNA triplex

## Abstract

This study explored the relationship between 3D genome organization and RNA–DNA triplex-forming sites of long noncoding RNAs (lncRNAs), a group of RNAs that do not code for proteins but are important factors regulating different aspects of genome activity. The triplex-forming sites of anti-sense cardiac lncRNA *GATA6-AS1* derived from DBD-Capture-Seq were examined and compared to modular features of 3D genome organization called topologically associated domains (TADs) obtained from Hi-C data. It was found that *GATA6-AS1* triplex-forming sites are positioned non-randomly in TADs and their boundaries. The triplex sites showed a preference for TAD boundaries over internal regions of TADs. Computational prediction analysis indicated that CTCF, the key protein involved in TAD specification, may interact with *GATA6-AS1*, and their binding sites correlate with each other. Examining locations of repeat elements in the genome suggests that the ability of lncRNA *GATA6-AS1* to form triplex sites with many genomic locations may be achieved by the rapid expansion of different repeat elements. Some of the triplex-forming sites were found to be positioned in regions that undergo dynamic chromatin organization events such as loss/gain of TAD boundaries during cardiac differentiation. These observed associations suggest that lncRNA–DNA triplex formation may contribute to the specification of TADs in 3D genome organization.

## 1. Introduction

Chromosomes are organized into modular units called topologically associated domains (TADs), which are separated by boundaries enriched in CCCTC-binding factor (CTCF) binding sites and highly transcribed genes [1,2,3]. Identifying factors and associated mechanisms that contribute to the specification and maintenance of TADs in 3D chromatin organization is essential for dissecting important biological processes. The “loop extrusion model” has also been suggested, as a linear tracking mechanism that involves CTCF and the Cohesin complex [4,5]. However, studies indicate that TAD formation may also depend on other factors [6,7,8]. One class of such factors that may contribute to the specification of TADs is the long noncoding RNAs (lncRNAs). LncRNAs are a group of RNAs that do not code for proteins and are usually longer than 200 nucleotides [9,10]. Thousands of RNA transcripts have been annotated as lncRNAs in humans and other species [11]. Once ignored as transcriptional noise, studies are uncovering that lncRNAs play important roles in many cellular processes. These include the regulation of nearby and distant genes, recruitment of chromatin modifiers, modulation of mRNA turnover, splicing and translation, and the formation and regulation of organelles and nuclear condensates [10,12]. In the context of the specification of TADs, the specific roles of lncRNAs have not been fully explored. Recently, an analysis that linked computationally predicted triplex-forming sites of lncRNAs with double-stranded DNA with features of TADs was reported [13]. Studies have reported that RNA–DNA triplex formation is one of the mechanisms used by lncRNAs to exert their functions. Upon its modulation, the triplex sites of lncRNA HOTAIR are enriched in differentially expressed genes and close to DNA-methylation changes [14]. LncRNAs such as MEG3 [15,16] and Fendrr [17] form triplex helices with DNA and transport protein complexes to specific regions of the genome to promote or repress gene expression [15,17]. In the context of TAD specification in 3D genome organization, the possibility of lncRNA–DNA triplex formation can be a mechanistically versatile mechanism to transport necessary factors or bring genomic regions closer.

This paper aims to investigate the role of lncRNA triplex-forming sites in the specification of TADs in 3D genome organization by analyzing and integrating two types of publicly available data sets: experimentally obtained genome-wide triplex-forming sites of lncRNA *GATA6-AS1* and Hi-C chromatin organization data during cardiac differentiation from two studies by Bertero et al. [18] and Kuo et al. [19], respectively. The study by Bertero et al. [18] investigated the 3D chromatin organization structure at different stages during cardiac differentiation (Figure S1A) of human embryonic stem cells. Out of 75 differentially expressed lncRNAs between day 0 and day 4 of cardiac differentiation, Kuo et al. [19] reported *GATA6-AS1* as the top-ranked cardiac lncRNA based on a ranking criterion that incorporates the triplex-forming potential, the number of triplex-forming sites, and the amount of fold change (Figure S1D,E). *GATA6-AS1* was also found to regulate mesoderm cardiac genes via triple helices and was essential for the differentiation of mesodermal cells into the cardiac mesoderm [19]. Therefore, *GATA6-AS1* serves as an ideal lncRNA candidate to investigate the role of lncRNA–DNA triplex-forming sites in mediating genome organization.

To explore the role of triplex-forming sites of *GATA6-AS1* in the specification of 3D genome organization, several analyses were performed. The occupancy of triplex-forming sites of *GATA6-AS1* was analyzed at specific locations of the 3D genome organization during cardiac differentiation. This revealed that *GATA6-AS1* triplex-forming sites were enriched in TAD boundaries, and TADs compared to randomly positioned genomic sites in the genome at different stages of cardiac differentiation. Interestingly, *GATA6-AS1* triplex-forming sites showed a preference for TAD boundaries compared to internal regions of TADs at different stages of cardiac differentiation. Since the genome goes through dynamic reorganization during lineage differentiation, the positions of the *GATA6-AS1* triplex sites were compared to dynamic events such as loss or gain of TAD boundaries during cardiac differentiation. Some triplex-forming sites of *GATA6-AS1* were positioned in genomic regions associated with loss and gain of TAD boundaries or switched between compartments A and B during cardiac differentiation. A potential association between *GATA6-AS1* sites and CTCF (one of the key players in TAD boundary specification) was also found. There was some evidence that *GATA6-AS1* may interact with CTCF and their binding sites are positioned in proximity to each other. Lastly, the origin of triplex-forming sites of *GATA6-AS1* because of expansion of repeat elements was investigated. At the genome-wide level, the existence of numerous *GATA6-AS1* triplex-forming sites may also be a result of the expansion of two kinds of repeat elements: LTRs and SINEs. However, LINEs and LINE-derived *GATA6-AS1* sites were under-represented at TAD boundaries. Conservation analysis reveals that *GATA6-AS1* triplex sites at TAD boundaries may be under a stronger selective constraint than those associated with non-TAD boundaries. With these results, preliminary cautious speculation suggests CTCF binds to *GATA6-AS1* and is transported to specific areas of the genome by *GATA6-AS1* via RNA–DNA triplex formation. This mechanism may contribute to the specification of TAD boundaries and other chromosomal contacts in spatial chromatin organization. It will be interesting to explore whether this mechanism is used in general by some triplex-forming lncRNAs to contribute to 3D genome specification.

## 2. Results

### 2.1. LncRNA GATA6-AS1-DNA Triplex Sites Are Enriched in TADs and TAD Boundaries and Prefer TAD Boundaries Compared to Internal Regions of TADs

Hierarchical units called topologically associated domains (TADs) are fundamental features of 3D genome organization. Understanding the mechanisms for the specification of TADs and their boundaries is necessary to decipher the relationship between the organization and function of the genome. Molecular factors such as CTCF and the Cohesin complex, which contribute to the specification of boundaries of topological associated domains, are preferentially enriched in TAD boundaries. A non-random positioning of *GATA6-AS1* triplex-forming sites in the context of 3D genome organization may indicate a role of *GATA6-AS1* triplex-forming sites in the specification of TADs and their boundaries.

**Figure 1:**
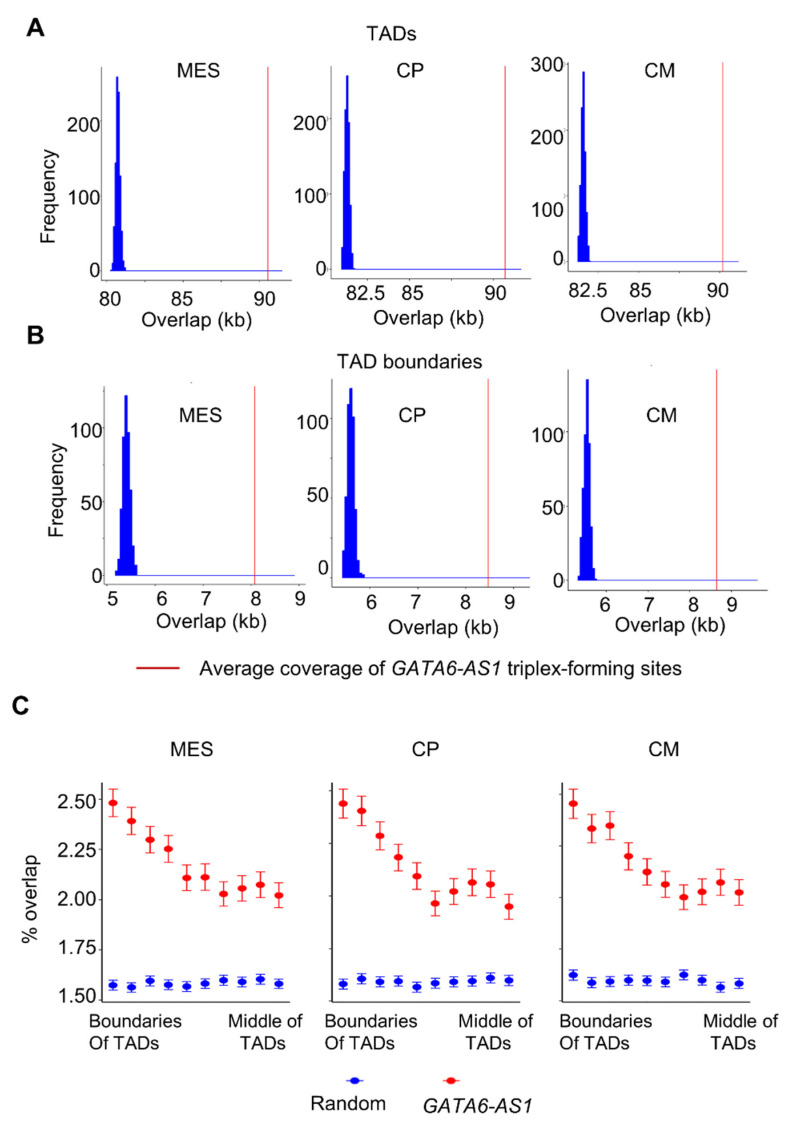
Enrichment of lncRNA *GATA6-AS1* triplex sites in specific regions in the context of 3D genome organization. The distribution of expected coverage (blue) compared to the observed coverage (vertical red line) in TAD domains and TAD boundaries are shown in panels (**A**,**B**), respectively. The *x*-axis and *y*-axis represent coverage (in kb) and frequency, respectively. (**C**) Enrichment of *GATA6-AS1* triplex sites at different regions in TADs. A TAD was divided into 10 regions or bins based on distance from the boundary. The *y*-axis represents the fraction of the size of the bin that was occupied by *GATA6-AS1* triplex sites (or a control set of randomized sites). The error bars indicate the standard error of the mean.

First, it was assessed whether the *GATA6-AS1* triplex-forming sites were enriched in the boundaries of TADs during cardiac differentiation. The observed coverage (or the number of base pair overlaps) of TADs with the triplex-forming sites of *GATA6-AS1* was computed. The observed coverage was compared against the distribution of expected coverage (Methods). The observed coverage of *GATA6-AS1* triplex-forming sites with TADs was significantly higher than the expected coverage (Figure 1A, *p*-value  <  10^−16^) at MES, CP, and CM stages during cardiac differentiation. Similarly, the observed coverage of *GATA6-AS1* triplex-forming sites with TADs was also significantly higher than the expected coverage (Figure 1B, *p*-value  <  10^−16^).

Next, it was determined whether there was a positional preference of the triplex-forming sites of *GATA6-AS1* at specific locations across the length of TADs. This can designate whether triplex-forming sites of *GATA6-AS1* prefer regions that are proximal or distant to the TAD boundaries. For this, each TAD was divided into 10 region/bins of equal length (Figure 1C) based on their distances from the TAD boundaries. The frequencies of triplex-forming sites in the bins were computed. The triplex-forming sites were positioned randomly within the entire genome and frequencies of randomly positioned regions in the 10 regions/bins were also computed. The triplex-forming sites were found to be unevenly distributed across the entire length of a TAD, displaying a preference for TAD boundaries over internal regions of TADs and showing significant deviation from the random control (Figure 1C, *p*-value  <  0.001 using the Kolmogorov–Smirnov test). This indicates a significant preference for triplex-forming sites of *GATA6-AS1* at regions proximal to TAD boundaries over regions that are distant from the boundaries.

### 2.2. Some of the GATA6-AS1-DNA Triplex Sites Are Positioned Nonrandomly in the Dynamic Rewiring of Genome Organization during Cardiac Differentiation

Dynamic rewiring of genome organization occurs during cardiac differentiation and is essential for the temporal regulation of the cardiac pathways. On a large genomic scale, it may involve the loss and gain of TAD boundaries or switching of genomic regions between the two types of compartments (A and B). Any possible role in the dynamic genome organization may reflect that *GATA6-AS1*-DNA triplex-forming sites are positioned in genomic regions which go through these dynamic chromatin organization events.

**Figure 2:**
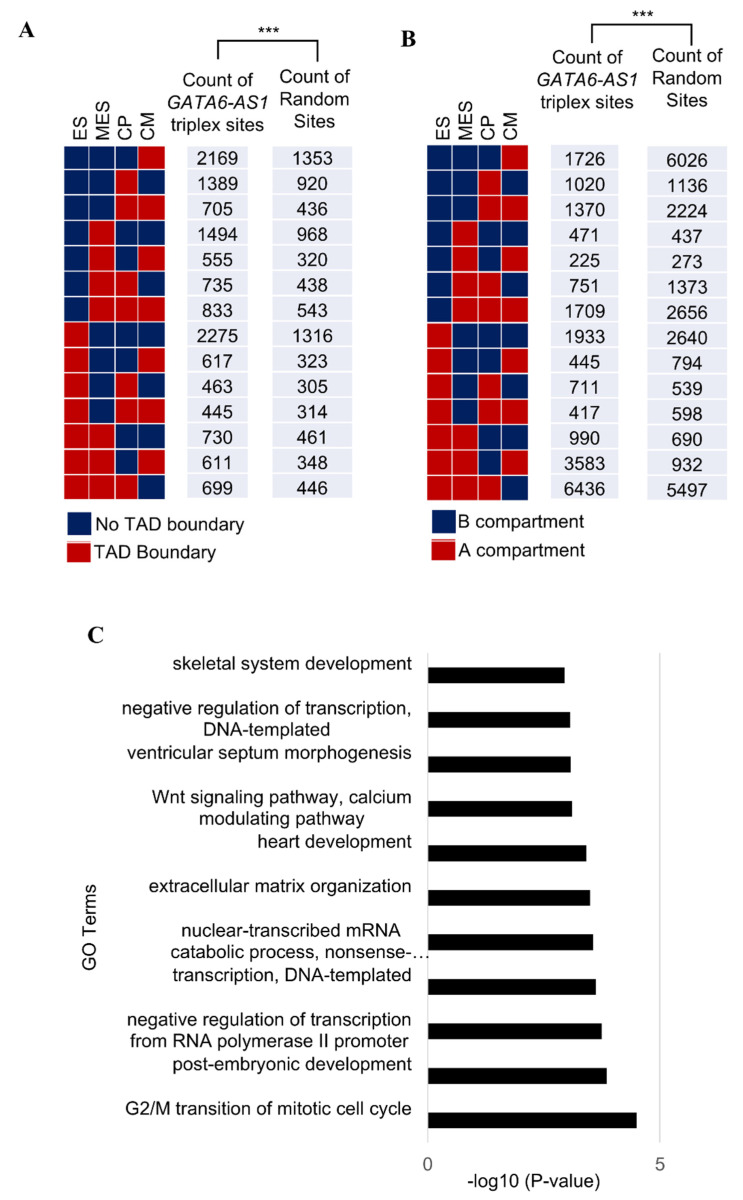
*GATA6-AS1* and dynamic loss/gain of TAD boundaries and A/B compartment switching. (**A**) Fourteen unique possibilities of a genomic site based on whether it was associated with the gain or loss of a TAD boundary during cardiac differentiation stages (ES, MES, CP, and CM). The counts for *GATA6-AS1* and the control set of randomized sites annotated as one of these unique possibilities are shown and were compared using the Chi-Square test. *p*-value < 0.001 is indicated by ***. (**B**) As in panel (**A**), 14 unique possibilities of a genomic site based on whether it was associated with switching between the two types of compartments, A and B, during the cardiac differentiation stages (ES, MES, CP, and CM). The counts for *GATA6-AS1* and the control set of randomized sites annotated as one of these unique possibilities are shown and were compared using the Chi-Square test. *p*-value < 0.001 is indicated by ***. (**C**) Gene ontology analysis of targets of triplex-forming sites of *GATA6-AS1*. The *x*-axis and *y*-axis show the top enriched GO “Biological Process” terms and −log10 *p*-value (enrichment score), respectively.

For this study, each triplex-forming site was annotated as one of 14 unique possibilities based on whether there was a loss or gain of TAD boundary (at least once) during the four stages of cardiac differentiation: the ES, MES, CP, or CM stage (Figure 2A). The number of *GATA6-AS1* triplex sites associated with these 14 possible annotations were computed. For comparison purposes, these triplex sites were randomly positioned in the genome and the expected counts of randomized sites for each of the 14 unique possibilities were also calculated. Overall, the distribution of the triplex-forming sites of *GATA6-AS1* was different from the expected distribution (*p*-value < 0.001 using the Chi-Square test). The same analysis was performed based on whether the triplex site occurred in a region that switched between the two types of compartments (A or B) during cardiac differentiation (Figure 2B). A significant difference was found between the distribution of the actual sites and the expected distribution (*p*-value < 0.001 using the Chi-Square test). These results show that some *GATA6-AS1* sites are positioned in genomic regions that undergo dynamic functional organization during cardiac differentiation.

Since *GATA6-AS1* had a peaked expression at the CP stage (Figure S1D), potential gene targets of *GATA6-AS1* sites during the CP stage were explored for any association with cardiac development. Gene targets of *GATA6-AS1* were determined by finding genes with transcription start sites closest to *GATA6-AS1* triplex sites. Gene ontology analysis on the target genes was performed using DAVID [20] with the entire human genome as the background. Enriched biological processes included terms associated with cardiac development such as “heart development”, “ventricular septum morphogenesis”, “Wnt signaling pathway”, “calcium modulating pathway”, etc. (Figure 2C). This may suggest a role of *GATA6-AS1* triplex sites in regulating the genes involved in cardiac development.

### 2.3. GATA6-AS1 May Interact with CTCF and Form Triplex Sites in Proximity to CTCF Binding Sites

**Figure 3:**
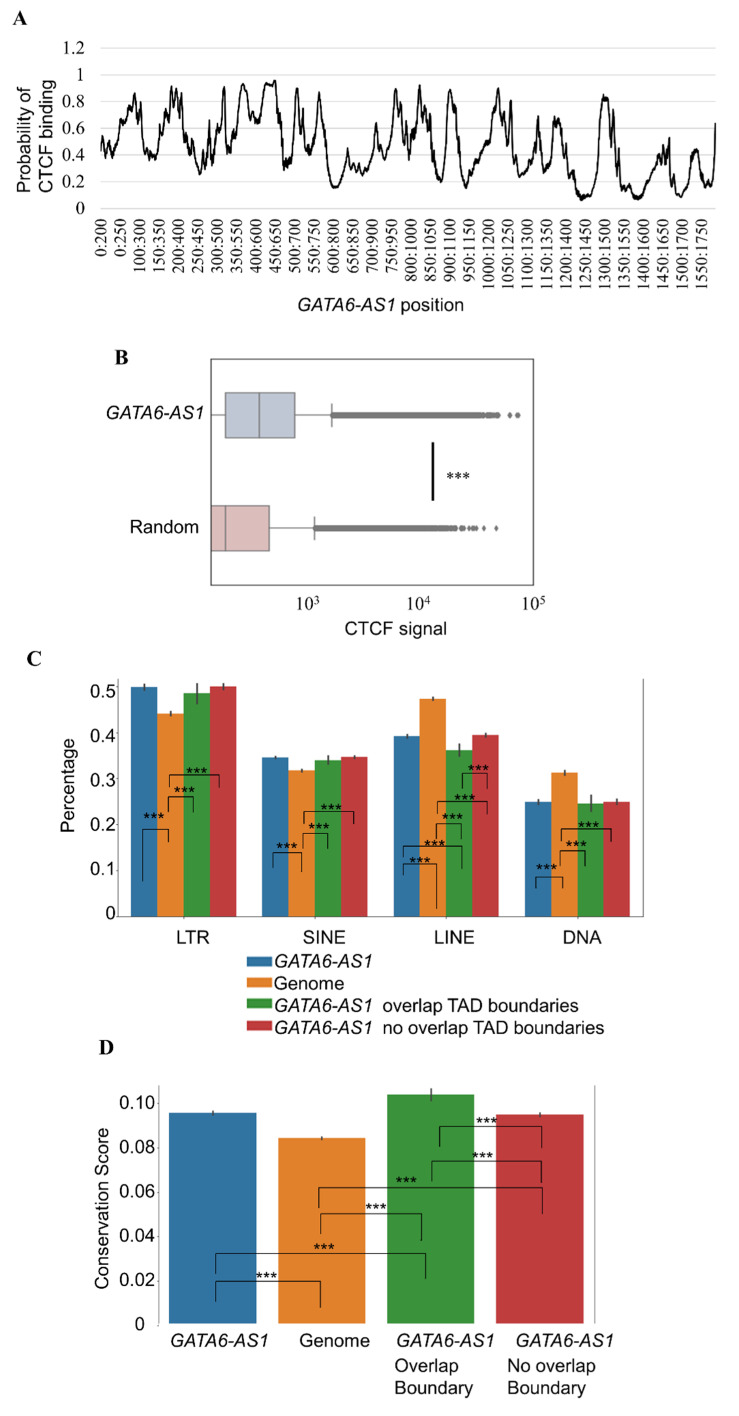
Characteristics of *GATA6-AS1* triplex sites in relationship to CTCF, repeat elements, and conservation. (**A**) The probability of binding of CTCF (*y*-axis) to different regions of the *GATA6-AS1* sequence (*x*-axis) is shown. (**B**) Boxplots comparing the amount of CTCF signal at *GATA6-AS1* triplex sites and a control set (genome). *** indicates *p*-value < 0.001. (**C**) Fraction (*y*-axis) of the length of *GATA6-AS1* triplex sites occupied by four groups of repeat elements (LTR, SINE, LINE, and DNA transposons) is compared to a control set (genome). *GATA6-AS1* triplex sites are further grouped into two groups based on whether it is associated with TAD boundaries or non-TAD boundaries. For each type of repeat element, six statistical comparisons were tested and only the ones which showed significant differences are marked as ***. (**D**) Conservation score per base pair (*y*-axis) is shown for *GATA6-AS1* triplex sites and a control set (genome). six statistical comparisons were tested and only the ones which showed significant differences are marked as ***.

CCCTC-binding factor (CTCF) has been shown to interact with several RNAs, and such interactions are essential for CTCF-mediated genome organization [21]. Recently, a deep learning tool called DeepLncCTCF [22] was developed to predict CTCF-RNA interaction accurately. The same tool was used to computationally predict the likelihood of an interaction of CTCF with *GATA6-AS1*. Several portions in the *GATA6-AS1* sequence had a high probability of interaction with CTCF (Figure 3A). Since *GATA6-AS1* most likely interacts with CTCF and also has triplex sites at various locations of the genome where CTCF is enriched such as TAD boundaries, *GATA6-AS1* may have a role in transporting CTCF to specified locations. To explore this possibility, the aggregated CTCF signals at the *GATA6-AS1* sites were computed and compared to the aggregated CTCF signals at random genomic sites (Figure 3B). The former was significantly higher than the latter (Figure 3B, *p*-value < 0.001, *t*-test), indicating the preferential enrichment of CTCF binding near triplex-forming sites of *GATA6-AS1*. This suggests that *GATA6-AS1* may interact with CTCF, transport it to specific locations, and contribute to CTCF-mediated chromatin organization during cardiac differentiation.

### 2.4. LINEs and LINE-Derived GATA6-AS1 Sites Are Under-Represented at TAD Boundaries

Triplex-forming sites of lncRNAs have been shown to coincide with the location of repeat elements [23], which may explain numerous binding sites of a single lncRNA in the genome. Therefore, *GATA6-AS1* triplex sites may be a consequence of repeat element expansion. To verify this, *GATA6-AS1* triplex sites were grouped based on whether they were located at TAD boundaries. Any potential differences were explored across the transposon classes that can derive the expansion of *GATA6-AS1* triplex sites at TAD boundaries during the CP stage. For each type of repeat element, the fraction of the length of the triplex sites that overlapped with SINEs, long terminal repeats (LTRs), long interspersed nuclear elements (LINEs), and DNA transposons were calculated (Figure 3C). LTR and SINE transposons were significantly overrepresented in *GATA6-AS1* triplex sites than in random sites (Figure 3C, *t*-test: *p*-value < 7.0 × 10^−53^), while LINE and DNA transposons were underrepresented (Figure 3C, *t*-test: *p*-value < 10^−56^). Occupancy of SINE, LTR, and DNA transposons in *GATA6-AS1* sites showed no significant preference between TAD boundaries or other regions (Figure 3C, *t*-test: *p*-value > 0.1). Lower LINE occupancy was observed in *GATA6-AS1* sites located at TAD boundaries compared to *GATA6-AS1* sites located in other regions (Figure 3C, *t*-test: *p*-value < 5.0 × 10^−6^). However, it was still lower than the LINE occupancy in random regions (Figure 3C, *t*-test: *p*-value < 5.0 × 10^−45^). This means only the LINE repeats overlapping with *GATA6-AS1* sites were underrepresented at TAD boundaries compared to non-TAD boundaries. This result is similar to the previous observation that LINE-derived CTCF sites appear under-represented at TAD boundaries and the insertion of long sequences such as LINEs is negatively selected at TAD boundaries [24].

Next, the evolutionary characteristics of *GATA6-AS1* triplex-forming sites at the CP stage were explored. For this, conservation scores from multiple alignments of 99 vertebrate genomes to the human genome were extracted at *GATA6-AS1* triplex sites (Methods). *GATA6-AS1* triplex sites were more conserved than random regions in the genome (Figure 3D, *t*-test: *p*-value < 2.0 × 10^−133^). Interestingly, the *GATA6-AS1* sites in TAD boundaries were more conserved than the triplex sites not associated with TAD boundaries (Figure 3D, *t*-test: *p*-value < 1.0 × 10^−11^). This indicates that *GATA6-AS1* triplex sites at TAD boundaries may be under a stronger selective constraint than those associated with non-TAD boundaries.

## 3. Discussion

In this paper, a computational analysis of the association between experimentally reported triplex-forming sites of lncRNA *GATA6-AS1* and 3D genome organization during cardiac differentiation is presented. The triplex-forming sites of *GATA6-AS1* are positioned nonrandomly in specific areas of the 3D genome organization, such as TADs and their boundaries. Compared to internal regions of TADs, TAD boundaries exhibit more triplex-forming sites of *GATA6-AS1*. A similar positional preference has been observed with other factors such as CTCF and the cohesion complex, which contribute to TADs specification [1,2,5,25]. Interestingly, a strong likelihood of interaction between CTCF with the *GATA6-AS1* sequence was computationally predicted, and proximities between CTCF binding locations and *GATA6-AS1* triplex sites were observed. Since previous studies have reported that CTCF-RNA interaction is necessary for the specification of CTCF-mediated 3D genome organization [21], it is likely that CTCF may bind to *GATA6-AS1* to be transported to specific locations such as TAD boundaries via a *GATA6-AS1*-DNA triplex-forming mechanism.

However, it is important to note that the observations in this paper do not prove a cause–effect relationship between *GATA6-AS1* triplex sites and TADs specification. Further experiments need to be performed to prove this hypothesis that the triplex-forming mechanism is essential for CTCF localization and ultimately the specification of TAD boundaries. For example, one can test the functional inhibition of triplex-formation and/or the triplex-forming domain of *GATA6-AS1* with DNA disrupts in CTCF binding and ultimately alter interactions in spatial chromatin organization. Some *GATA6-AS1* triplex-forming sites were found to be located in regions associated with the gain or loss of a TAD boundary during cardiac differentiation. In general, developmental biological processes involve the dynamic rewiring of chromatin interactions and organization [2,7,18], which require the localization of CTCF at appropriate locations at different stages of the process for the specification of correct CTCF-mediated chromatin loops/interactions. These rewiring events may be dependent on lncRNAs (such as *GATA6-AS1*) that interact with CTCF and exhibit stage-specific expression patterns during these biological developmental processes. The transportation of CTCF to specific locations for the dynamic rewiring of genome organization can be achieved by these types of lncRNAs.

For the first time, an association between the experimentally obtained genome-wide lncRNA–DNA triplex sites and 3D genome specification is reported in this paper. Such an analysis is possible because of the availability of the experimentally obtained genome-wide triplex-forming sites of *GATA6-AS1* and TAD locations at different stages of cardiac differentiation. However, it has also been reported by multiple studies that some lncRNAs can form triplex sites with the DNA to modulate gene expression [14,17]. Therefore, it is highly likely that there are lncRNAs such as *GATA6-AS1* with elevated levels of expression, that form RNA–DNA triplex structures during the differentiation of different lineages [26]. To concretely confirm and validate results during the differentiation of different lineages will require additional experiments such as a genome-wide DBD-capture-Seq of selected lncRNAs, which is beyond the scope of this paper. This will be pursued in further studies. However, to explore other lncRNAs in cardiac differentiation, 32 lncRNAs reported by Kuo et al. [19] with a fold change of greater than two at the CP stage compared to day 0 of cardiac differentiation were examined (Supplementary Table S1). Out of these 32 lncRNAs, only 14 were reported to have a triplex-forming potential by Kuo et al. [19]. This suggests that the triplex-forming mechanism may not be used by all lncRNAs with elevated expressions during cardiac differentiation. Except for *GATA6-AS1*, experimentally obtained triplex-forming sites of other lncRNAs were not reported [19]. Because of this, triplex-forming sites for the other 13 lncRNAs were predicted using the triplexator tool [27]. Two lncRNAs (N4BP2L2-IT2 and ZNF436-AS1) were ignored because of a very small number of triplex-forming sites. All lncRNAs, except BAALC-AS1, ID2-AS1, and KC6 in TAD boundaries, showed a positional preference in TAD boundaries and TADs (Supplementary Figures S2 and S3). These results strongly indicate that some lncRNAs with elevated expression during cardiac differentiation may use the triplex-forming mechanism to mediate chromatin organization. However, these lncRNAs may contribute to chromatin organization via triplex formation to different degrees. Kuo et al. [19] reported that *GATA6-AS1* was the top-ranked lncRNA (Supplementary Table S1) based on the number of triplex-forming sites, size of triplex-forming domain in the lncRNAs sequences, and amount of elevated expression. Nevertheless, the results suggest the triplex-forming mechanism may be a general mechanism used by some lncRNAs to mediate chromatin organization. Like a transcription factor that interacts with many genomic sites to modulate different genes [28], a single lncRNA has the potential to form triplex sites at multiple locations of the genome, contributing to the specification of multiple TADs. The formation of multiple triplex sites by lncRNA is most likely achieved by the expansion of repeat elements in the genome. Even though there are many transcription factors involved during the differentiation of a specific lineage, the specification of a lineage is primarily dependent on a small number of master transcription factors [28]. Similarly, the specification of many TADs during the differentiation of a lineage may be dependent on the triplex-forming sites of a handful of lncRNAs. The ability of some lncRNAs to target different sites of the genome via RNA–DNA triplex formation to transport molecules such as CTCF may be a versatile mechanism involved in the specification of TADs in 3D genome organization. This paper presents a link between a specific lncRNA’s triplex-forming sites and 3D genome organization, thus indicating another layer of 3D genome regulation by lncRNAs via RNA–DNA triplex formation.

## 4. Materials and Methods

### 4.1. Enrichment Analysis of GATA6-AS1 Triples Sites at Specific Regions of the 3D Genome

The study by Bertero et al. [18] investigated the 3D chromatin organization structure at different stages during the cardiac differentiation (Figure S1A) of human embryonic stem cells. From this study, Hi-C data and TAD locations (Figure S1B) were acquired from the GEO repository (NCBI GEO ID: GSE106690) at days 0 (hESC), 2 (Mesoderm or MES), 5 (Cardiac Progenitors or CP), and 14 (cardiomyocytes or CM) during the cardiac differentiation. From the study by Kuo et al. [19], the genome-wide triplex-forming sites of lncRNA *GATA6-AS1* (NCBI GEO ID: GSE119638) were obtained. These sites were generated using an RNA-based DNA capture assay (DBD-Capture-Seq) on day 4, during cardiac differentiation (Figure S1C) using human pluripotent cells (hPSCs).

To assess whether the triplex-forming sites of *GATA6-AS1* are enriched in regions of interest (TADs or boundaries of TADs), the observed coverage (or the number of base pair overlaps) of regions of interest with the triplex-forming sites was computed using bedtools [29]. A control set of genomic regions was generated by randomly positioning the real *GATA6-AS1* triplex sites within the genome. An expected coverage was computed as the coverage of these randomized control sites with the regions of interest. This random shuffling was performed 1000 times for each shuffled set; an expected coverage was obtained. Normal distributions were estimated to fit the distribution of the expected coverages. The *p*-values were computed using the observed coverage in the region of interest and estimated parameters of the normal distribution.

To compare the differential positional preference of the *GATA6-AS1* triplex-forming sites between the TADs boundaries and regions within the TADs, the positions of *GATA6-AS1* triplex-forming sites that overlap with boundaries of TADs were randomly positioned so that they fell only within the TADs (excluding the boundary regions). Each TAD was divided into 10 regions of equal length depending on the distance from the TAD boundary. TADs are not all of the same size. Therefore, when each TAD is divided into 10 bins, bins from larger TADs are larger and are expected to harbor more triplex-forming sites. Therefore, for each bin across all TADs, the fraction of the length of the bin that was occupied by *GATA6-AS1* triplex sites was computed. For each bin, the mean value and standard error of the mean (SEM) were computed from all the TADs. The same was performed with the randomly positioned sites.

### 4.2. Analysis of GATA6-AS1 Triples Sites in the Context of Dynamic Rewiring Chromatin during Cardiac Differentiation

The *GATA6-AS1* sites were analyzed in the context of large-scale dynamic rewiring of the chromatin during cardiac differentiation. Fourteen unique groups were generated based on whether a triplex site was positioned in a genomic region associated with the loss or gain of the TAD boundary during the four stages (day0, MES, CP, and CM) in cardiac differentiation. The number of *GATA6-AS1* triplex sites in 14 groups was computed. Similar counts were computed for a control set of randomly positioned sites. The two distributions were compared using the Chi-square test. A similar analysis was also performed by examining whether the *GATA6-AS1* triplex sites were in regions that underwent switching of compartments between types A and B.

### 4.3. GO Analysis

Gene ontology analysis was performed for the enrichment of terms related to “Biological Processes” using DAVID [20].

### 4.4. Prediction of CTCF-Binding

The prediction of the likelihood of CTCF binding to *GATA6-AS1* was performed using DeepLncCTCF [22]. All subsequences of length 201 from *GATA6-AS1* were fed to DeepLncCTCF to obtain the probability of CTCF binding to these sequences.

### 4.5. Repeat Element Analysis

The locations of repeats were downloaded from the UCSC genome browser [30] for the hg38 genome version. Using bedtools [29], all *GATA6-AS1* triplex sites (at CP stage of cardiac differentiation) that overlapped with four groups of repeat elements (LTR, LINE, SINE, and DNA transposons) were extracted. For such overlapping *GATA6-AS1* sites, the fraction of the length of the site occupied by repeat elements was computed using the tool bigWigAverageOverBed [30]. The *GATA6-AS1* sites were assigned to one of two groups based on whether they were associated with TAD boundaries. A control set was generated by randomly positioning the *GATA6-AS1* triplex sites in the genome. The fractions of the four groups of repeat elements were computed in the control set. For each type of repeat element, four bar plots were generated which represent the occupancy of a repeat element in all *GATA6-AS1* sites, *GATA6-AS1* sites located at TAD boundaries, *GATA6-AS1* sites located at non-TAD boundaries, and a control set.

### 4.6. Conservation Scores

To perform a conservation analysis of *GATA6-AS1* sites, the conservation scores (“phast cons”) for alignments of 99 vertebrate genomes with the human genome (hg38) were acquired from the UCSC genome browser [30]. As with the analysis of repeat elements, four groups of sites were used: *GATA6-AS1* triplex sites, *GATA6-AS1* triplex sites located at TAD boundaries, *GATA6-AS1* sites located at non-TAD boundaries, and a control set. Using the bigWigAverageOverBed tool [30], the conservation score per base pair in each site of these groups was computed. Pairwise comparisons were performed between these groups using a pairwise *t*-test. All these analyses were performed for the CP stage of cardiac differentiation.

### 4.7. Triplex-Forming Sites of Other Cardiac lncRNAs

The tool triplexator was used to predict the triplex sites of other cardiac lncRNAs with the following options: -l 20 -fr off -c 2 of 0. The sequences of these lncRNAs were obtained from the GENCODE database (gencodegenes.org, accessed on 11 April 2022). All isoforms of the same lncRNA were used. Overlapping triplex-forming sites were merged into a single site.

## Data Availability

Locations of *GATA6-AS1* triplex sites and TADs are available in NCBI Gene Expression Omnibus (GEO) repository (https://www.ncbi.nlm.nih.gov/geo/) with IDs GSE106690 and GSE119638, respectively (accessed on 10 May 2021).

## Supplementary Materials

The following supporting information can be downloaded at: https://www.mdpi.com/article/10.3390/ncrna8030041/s1, Figure S1: LncRNA *GATA6-AS1* in cardiac differentiation from previous studies; Figure S2: Enrichment of 9 cardiac lncRNAs’ triplex sites in TAD boundaries at CP stage; Figure S3: Enrichment of 9 cardiac lncRNAs’ triplex sites in TADs at CP stage; Table S1: LncRNAs with fold change >2 at CP stage compared to day 0 of cardiac differentiation reported by Kuo et al.
