# Supplementary material for: Association between Triplex-Forming Sites of Cardiac Long Noncoding RNA GATA6-AS1 and Chromatin Organization
Source: Noncoding RNA. 2022 Jun 1;8(3):41. doi: 10.3390/ncrna8030041 (PMC9227037; doi:10.3390/ncrna8030041)
Supplement: Supplementary file 1 [file ncrna-08-00041-s001.zip › ncrna-1629845-supplementary.pdf]

Association between Triplex-Forming Sites of Cardiac Long Noncoding RNA GATA6-AS1 and Chromatin Organization

Supplementary Data

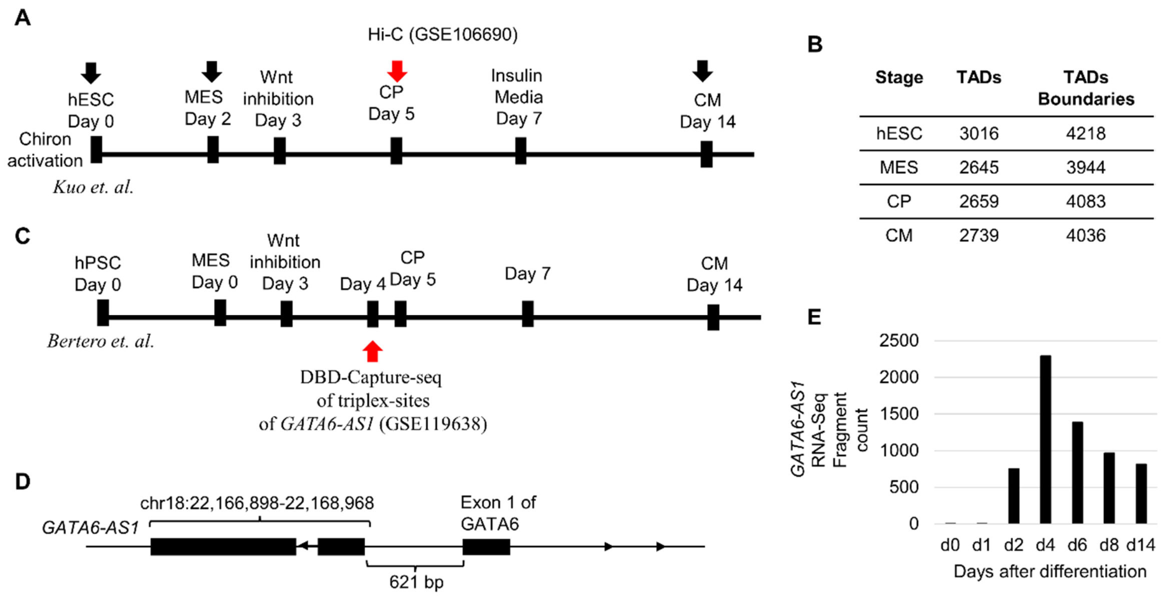

Figure S1. LncRNA *GATA6-AS1* in cardiac differentiation from previous studies. (**A**) Different Stages of cardiac differentiation with Hi-C data from the study by Kuo et. al. [19] (**B**) Number of TAD and TAD boundaries at different stages of cardiac differentiation from panel (**A**). (**C**) DBD-capture-seq data was performed to obtain triplex-forming sites of lncRNA GATA6-AS1 at day 5 after differentiation in a study by Bertero et. al [18]. (**D**) Genomic location of GATA6-AS1 relative to protein-coding gene GATA6. (**E**) Expression profile of GATA6-AS1 at different stages of cardiac differentiation.

Table S1: LncRNAs with fold change > 2 at CP stage compared to day 0 of cardiac differentiation reported by Kuo et al. [19]. The lncRNAs are provided in order of their rank (highest rank first) assigned by Kuo et al. [19] based on the number of triplex-forming sites, size of triplex-forming domain in the lncRNAs sequences, and amount of elevated expression.

**LncRNA**	**Fold Change**	**Triplex-forming potential from** Kuo et al. [19]
GATA6-AS1	6.22	Yes
JHDM1D-AS1 or KDM7A-DT	3.51	Yes
HOXB-AS1	7.34	Yes
LINC00261	4.87	Yes
KC6	4.41	Yes
SEC24B-AS1	2.72	Yes
ID2-AS1	3.73	Yes
MALAT1	3.06	Yes
HAND2-AS1	7.32	Yes
LINC00890 or SERTM2 (protein-coding gene)	4.43	Yes
ZNF436-AS1	4.35	Yes
MEF2C-AS1	4.05	Yes
LINC00894 or EOLA2-DT	3.64	Yes
BAALC-AS1	2.17	Yes
N4BP2L2-IT2	2.2	Yes
TMCC1-AS1	3.33	No
DICER1-AS1	2.37	No
MANEA-AS1	2.57	No
LINC01473	3.37	No
ACVR2B-AS1	2.35	No
LINC01125	2.28	No
GATA3-AS1	5.56	No
LINC01099	6.91	No
TBX2-AS1	7.46	No
LINC01440	6.02	No
LINC00648	4.05	No
ALOX12-AS1	2.41	No
TAPT1-AS1	2.69	No
CAHM	2.04	No
MIR210HG	2.37	No
VIM-AS1	2.29	No
PTOV1-AS1	2.23	No
MFI2-AS1	2.28	No

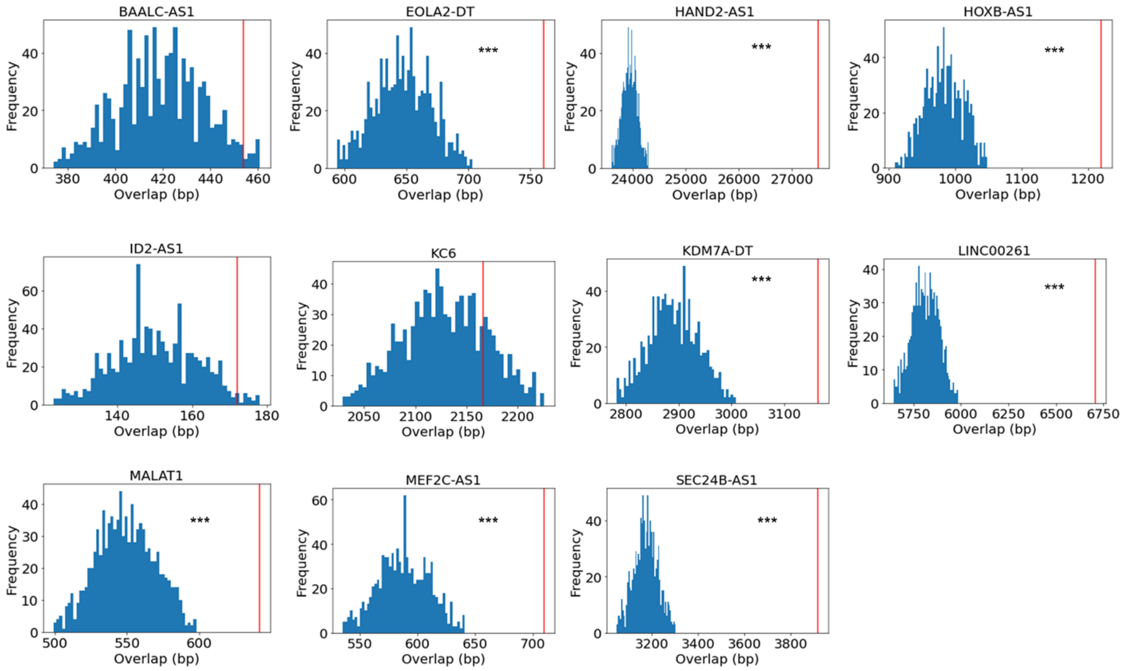

Figure S2. Enrichment of 9 cardiac lncRNAs' triplex sites in TAD boundaries at CP stage. The distribution of expected coverage (blue) compared to the observed coverage (vertical red line) in TAD boundaries are shown. The x-axis and y-axis represent coverage, and frequency, respectively. A statistical difference between expected coverage and random measurements is indicated by *** (p-value < 0.001).

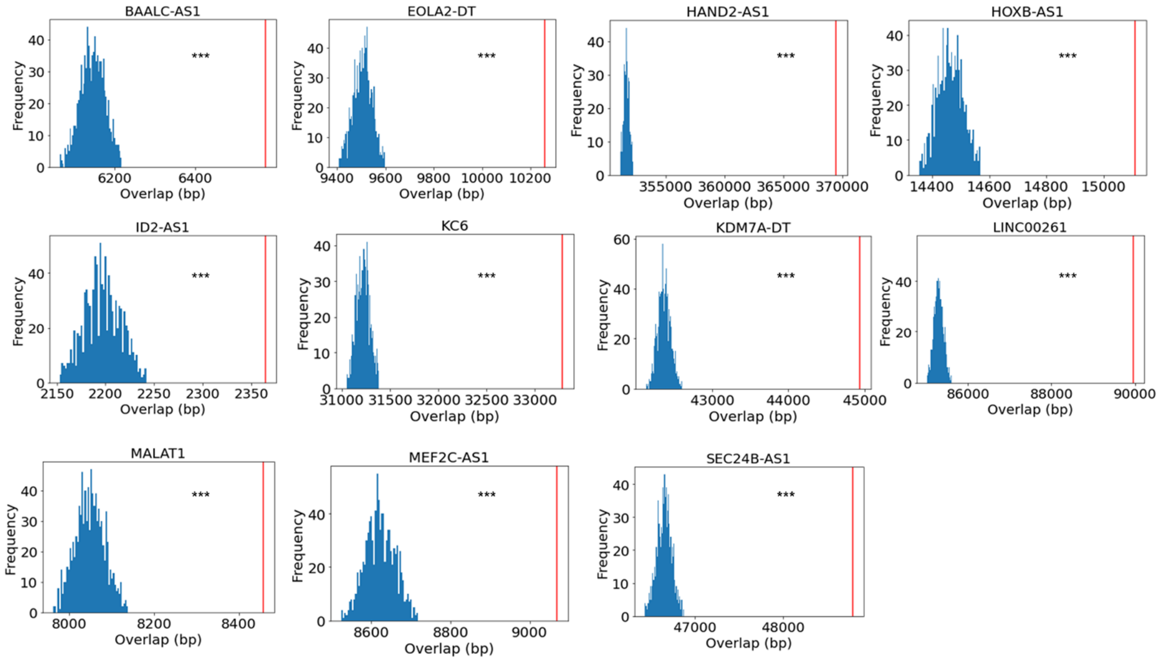

Figure S3. Enrichment of 9 cardiac lncRNAs' triplex sites in TADs at CP stage. The distribution of expected coverage (blue) compared to the observed coverage (vertical red line) in TAD domains are shown. The x-axis and y-axis represent coverage, and frequency, respectively. A statistical difference between expected coverage and random measurements is indicated by *** (p-value < 0.001).

References

18 Bertero, A.; Fields, P.A.; Ramani, V.; Bonora, G.; Yardimci, G.G.; Reinecke, H.; Pabon, L.; Noble, W.S.; Shendure, J.; Murry, C.E. Dynamics of genome reorganization during human cardiogenesis reveal an RBM20-dependent splicing factory. *Nat. Commun.* **2019**, *10*, 1538. https://doi.org/10.1038/s41467-019-09483-5.

19 Kuo, C.C.; Hänzelmann, S.; Sentürk Cetin, N.; Frank, S.; Zajzon, B.; Derks, J.P.; Akhade, V.S.; Ahuja, G.; Kanduri, C.; Grummt, I.; et al. Detection of RNA-DNA binding sites in long noncoding RNAs. *Nucleic Acids Res.* **2019**, *47*, e32. https://doi.org/10.1093/nar/gkz037.
